# Comparison of Lung Inflammatory and Transcriptional Responses in Mice and Rats Following Pulmonary Exposure to a Fiber Paradigm-Compatible and Non-Compatible MWCNT

**DOI:** 10.3390/nano15171364

**Published:** 2025-09-04

**Authors:** Laura Aliisa Saarimäki, Pernille Høgh Danielsen, Kristina Bram Knudsen, Sarah Søs Poulsen, Sabina Halappanavar, Henrik Wolff, Pia Anneli Sofia Kinaret, Dario Greco, Ulla Vogel

**Affiliations:** 1 Finnish Hub for Development and Validation of Integrated Approaches (FHAIVE), Faculty of Medicine and Health Technology, Tampere University, 33520 Tampere, Finland; 2 Division of Pharmaceutical Biosciences, Faculty of Pharmacy, University of Helsinki, 00790 Helsinki, Finland; 3 National Research Centre for the Working Environment, DK-2100 Copenhagen, Denmark; 4 Environmental Health Science and Research Bureau, Health Canada, Ottawa, ON K1A0K9, Canada; 5 Department of Biology, University of Ottawa, Ottawa, ON K1N6N5, Canada; 6 Laboratory of Pathology, Finnish Institute of Occupational Health, 00250 Helsinki, Finland; 7 Institute of Biotechnology, University of Helsinki, 00790 Helsinki, Finland; 8 National Food Institute, Technical University of Denmark, DK-2800 Kgs. Lyngby, Denmark

**Keywords:** toxicology, inflammation, acute phase response, fibrosis, multi-walled carbon nanotubes, transcriptomic, KEGG pathway enrichment, pulmonary exposure, animal models

## Abstract

Inhalation of multi-walled carbon nanotubes (MWCNTs) poses potential health risks due to their structural similarity to asbestos and their ability to induce chronic lung inflammation, fibrosis, and lung cancer in animal models. This study investigated the pulmonary inflammatory and transcriptomic responses of two distinct MWCNTs—NM-401 (long, rigid) and NM-403 (short, thin)—in rats and mice using intratracheal instillation at matched dose levels at two post-exposure time points. Both MWCNTs induced acute neutrophilic inflammation and dose-dependent transcriptomic alterations in both species, with NM-403 eliciting a stronger response. Transcriptomic profiling revealed a substantial overlap in differentially expressed genes across materials and species, particularly at the early time point. Fibrosis-associated genes were upregulated in both species, with more persistent expression observed in rats. Acute phase response genes, including *Orosomucoid 1* and *Lipocalin 2* were commonly induced, while *Serum Amyloid A3* and *Orosomucoid 2* were selectively upregulated in mice. Functional enrichment analyses showed conserved activation of immune and inflammatory pathways. Our findings show that even short, non-fiber-like MWCNTs can provoke potent and persistent pulmonary effects, challenging assumptions based solely on MWCNT properties. Despite differences in long-term responses, the overall inflammatory and transcriptional profiles showed strong interspecies concordance, suggesting that both rats and mice are relevant models for assessing MWCNT-induced pulmonary toxicity.

## 1. Introduction

Multi-walled carbon nanotubes (MWCNTs) are high-aspect-ratio nanomaterials with many technological applications due to their unique structural and mechanical properties [1]. However, concerns have been raised about possible health risks after inhalation exposure, particularly in occupational settings. These concerns stem from their similarity to asbestos and other fibers with asbestos-like effects, as well as from animal studies demonstrating their toxicity. Inhalation or pulmonary administration of carbon nanotubes (CNTs) has been shown to induce chronic inflammation, fibrosis, genotoxicity, plaque progression, acute-phase responses, and lung cancer in rodents [2,3,4,5,6,7,8,9,10,11].

MWCNTs vary greatly in their physicochemical properties, such as length, diameter, rigidity, and surface characteristics [12,13]. Their toxicity may depend on these properties; for example, animal studies show that varying metal content can affect neutrophil influx and the acute-phase response [13]. Moreover, studies indicate that surface modifications, such as adding chemical functional groups or shortening the fibers, can reduce genotoxicity and pulmonary inflammation [14,15,16]. Understanding the mechanisms underlying MWCNT-induced adverse effects through approaches like the Adverse Outcome Pathways (AOP) model [17,18], and identifying key physicochemical determinants [8,9], are critical for risk assessment and for developing safe-by-design strategies.

Both inhalation and pulmonary administration of CNTs trigger strong and persistent inflammatory responses in rats and mice [2,13,19,20]. However, important species differences exist. Rats are more prone to CNT-induced fibrosis and lung cancer [2,3,10,11,21,22,23], whereas mice typically develop less fibrosis. In contrast to rats, mice show robust upregulation of the acute-phase proteins serum amyloid A (SAA) isoforms (*Saa1*, *Saa2*, *Saa3*), following pulmonary CNT exposure [9,24,25,26,27,28]. Since SAA plays a central role in atherosclerotic plaque progression, this mouse-specific response strengthens the mechanistic link between CNT exposure and cardiovascular disease risk [5,24,25,26,27].

One of the most toxic MWCNTs is Mitsui-7, a thick and rigid nanotube that induces lung cancer in rats after inhalation exposure [3]. According to the fiber paradigm (FP)—a classification for high-aspect-ratio fibers with carcinogenic potential [28]—the European Chemicals Agency (ECHA) Risk Assessment Committee (RAC) has classified FP-compatible MWCNTs (diameter between 30 nm and 3 μm, length ≥ 5 μm, aspect ratio ≥ 3:1) as category 1B carcinogens indicating sufficient evidence in animals, though human evidence remains limited [29]. MWCNTs can broadly be divided into FP-compatible and non-compatible types. FP-compatible materials, such as Mitsui-7 and NM-401, are long, rigid, and biopersistent. These properties allow them to interact with lung tissue much like asbestos fibers. In contrast, non-compatible MWCNTs, such as NM-403, are shorter, more entangled, and flexible. Such morphology reduces their potential to act as pathogenic fibers and is generally associated with lower fibrogenic potency, although they may still trigger significant acute inflammatory responses [22]. On the other hand, short and thin MWCNTs induce more surface area-dependent inflammation and acute-phase responses [16]. Notably, both CNT types induce lung adenomas in rats with equal potency following pulmonary dosing [3,10,11,30]. Recent studies indicate that NM-403 can induce persistent lung inflammation and epithelial hyperplasia, but through mechanisms distinct from Mitsui-7 [30,31]. In contrast, our previous studies demonstrated that NM-401 induces interstitial fibrosis in both rats and mice, whereas NM-403 does not [21,22,32]. Interestingly, NM-403 exposure in rats has been associated with pulmonary alveolar proteinosis, a condition usually associated with quartz exposure [22].

Given the wide use of rodent models in risk assessment and nanotoxicology, this study compares pulmonary transcriptional responses in mice and rats exposured to two MWCNTs with distinct physicochemical properties. NM-401 is long, thick, and needle-like, and thus fiber-compatible. NM-403 in contrast, is too short and thin to meet the FP. We evaluate inflammation by assessing neutrophil influx, perform transcriptomic pathway-level analyses, and compare gene-level responses using predefined transcriptional signatures for fibrosis and acute-phase responses.

## 2. Materials and Methods

### 2.1. Multi-Walled Carbon Nanotubes

Pristine MWCNTs, NM-401 and NM-403, were obtained from the Joint Research Centre (Ispra, Italy). Their physicochemical properties have been extensively characterized previously [12,22,32] and are summarized in Table 1. According to published transmission electron microscopy (TEM) images, the main difference between the two MWCNTs is the physical dimensions (dimeter and length) as well as the level of nanotube aggregation: NM-401 mainly appears as individual fibers of varying length with occasional small entanglements, whereas the shorter and thinner NM-403 forms large, highly entangled agglomerated bundles [22]. Overall, NM-401 possesses physicochemical properties similar to Mitsui-7, an MWCNT known to induce lung tumors in rats following inhalation exposure [3], and is classified as possibly carcinogenic to humans [30,33]. Other short and thin MWCNTs have been shown to induce dose-dependent fibrosis in sub-chronic inhalation studies [2]. The specific surface area of NM-401 is reported as 18 m^2^/g, whereas NM-403 has a significantly higher surface area of 135 m^2^/g—approximately 7.5 times larger (Table 1).

Mice and rats were exposed to NM-401 or NM-403, with matched µg/body weight doses between species. Mice received either 18 or 54 µg per animal, and rats 180 or 540 µg per animal. This corresponds to 0.966 and 2.897 mg/kg bw for mice, and 0.959 and 2.877 mg/kg bw for rats, respectively (Table 2). When accounting for surface area, the 7.5-fold difference between NM-401 and NM-403 translated to approximately a 3-fold difference in deposited surface area doses between the two MWCNTs (Table 3).

### 2.2. Animals and Exposure

Exposure protocols for mice and rats were previously described [19,21,22]. Female Sprague Dawley rats (7–8 weeks old) and female C57BL/6 mice (6–7 weeks old) were obtained from Taconic Europe (Ejby, Lille Skensved, Denmark). We used females to ensure continuity which allows comparison and benchmarking with our prior inhalation and instillation studies, thereby facilitating direct comparisons of dose–response, transcriptomic profiles and other outcomes across projects. In addition, female mice are less aggressive than males and can be housed in groups of 6 per cage, which reduces stress and improves logistical feasibility. Animals were randomly allocated to experimental groups (6 per cage for mice and 2 per cage for rats) and acclimatized for at least one week prior to exposure. They were housed in polypropylene cages with sawdust bedding and environmental enrichment under controlled conditions (temperature 21 ± 1 °C, relative humidity (50 ± 10%), and a 12 h light/dark cycle). Food and tap water were provided ad libitum. Animals were monitored daily for general health, as previously described [34]. All procedures compiled with EC Directive 2010/63/EU and received approval from the Danish Animal Experiment Inspectorate (permit no. 2006/561-1123, approved 11 April 2006 and no. 2010/561-1779, approved 23 March 2010).

Rodents received intratracheal instillations of NM-401 or NM-403 at doses of 18 or 54 µg per mouse, and 180 or 540 µg per rat. These doses corresponded to 0.966 and 2.897 mg/kg body weight for mice and 0.959 and 2.877 mg/kg for rats, respectively. The administered doses (180 or 540 µg/rat; 18 or 54 µg/mouse) approximate one- and three-fold the estimated cumulative lung burden from 40 years of occupational exposure at the NIOSH-recommended exposure limit of 1 µg carbon/m^3^ [35]. This estimate considered a 10% pulmonary deposition fraction [20], species-specific ventilation rates (1.8 L/h for mice, 18 L/h for rats), and a 40 h work week [8,22]. The intratracheal instillation followed established in-house procedures [36,37]. Briefly, animals were anesthetized with 4% Isoflurane and instilled through the trachea using a 24 gauge catheter with a shortened needle for the mice and a 1 mL plastic syringe for rats [36,38]. Suspensions of MWCNTs were prepared in NanoPure water containing 2% homologous serum to ensure stable, reproducible suspensions while minimizing agglomeration and dispersed by sonication [39]. Control animals were administered the vehicle suspension. Mice received 50 µL of the suspension; rats received 400 µL. Animals were monitored for health status during the experiment and were euthanized at 1 and 28 days post-exposure. Upon termination, mice were anesthetized by subcutaneous injection of a ZRF cocktail (Zolazepam 3.29 mg/mL, Tiletamine 3.29 mg/mL, Xylazine 0.45 mg/mL, and Fentanyl 2.6 µg/mL in sterile isotone saline; dose: 0.1 mL/10 g bodyweight). Rats received subcutaneous injection of Zoletil Forte (250 mg/mL), Rompun (20 mg/mL), and Fentanyl (50 µg/mL) in sterile isotone saline (dose: 1 mL/250 g body weight). Following anesthesia, euthanasia was completed by withdrawal of heart blood.

### 2.3. Sample Collection

Bronchoalveolar lavage (BAL) fluid was collected as described previously [34]. Lungs were flushed via the trachea—twice with 5 mL of sterile 0.9% NaCl in rats and 1 mL in mice. The total cell number was determined using a NucleoCounter live/dead assay (ChemoMetec, Lillerød, Denmark), and cytospins were prepared using 50 µL of suspension. Lung tissues were sectioned, snap-frozen in liquid nitrogen, and stored at −80 °C for total RNA extraction.

### 2.4. Bronchoalveolar Lavage Cell Composition

BAL cell composition was determined as previously described [34]. Briefly, 50 μL of cell suspension was centrifuged onto microscope slides at 10,000 rpm for 4 min using a Cytofuge 2 (StatSpin, TRIOLAB, Brønby, Denmark). Slides were fixed in 96% ethanol and stained with May-Grünwald-Giemsa stain. Two hundred cells per sample were counted to determine differential counts.

### 2.5. RNA Extraction and Quality Assessment

RNA was extracted from lung tissue using TRIzol (Thermo Fisher Scientific, Carlsbad, CA, USA), treated with DNAse, and purified with RNeasy Mini Kit (Qiagen, Aarhus, Denmark) according to the manufacturer instructions. RNA integrity was verified with a Bioanalyzer (Agilent Technologies, Santa Clara, CA, USA); samples with RNA Integrity Number (RIN) ≥ 7.0 were used for the microarray analysis.

### 2.6. Microarray Hybridization

Microarray experiments were conducted using the Agilent Two-Color Microarray-Based Gene Expression Analysis protocol (version 6.9.1, Agilent Technologies, Santa Clara, CA, USA). Briefly, 150 ng of total RNA from each sample was primed and converted to cDNA, which was then transcribed into cRNA, labeled with either Cy3 or Cy5, and amplified using the Low Input Quick Labeling Kit (Agilent Technologies, Santa Clara, CA, USA). The labeled cRNA was purified with the RNeasy Mini Kit (Qiagen). The concentration of cRNA and the fluorescent dyes (Cy3 or Cy5) were measured using a NanoDrop ND-2000 spectrophotometer (Thermo Fisher Scientific, Carlsbad, CA, USA). These values were further used to calculate the yield and specific activity (concentration of dye in pmol/µL divided by the concentration of cRNA in ng/µL multiplied by 1000) to ensure the manufacturer’s recommended limits were met (>0.825 µg of cRNA and specific activity > 6). For hybridization, 300 ng of Cy3-labeled cRNA was combined with a corresponding Cy5-labeled sample, fragmented, and hybridized onto Agilent SurePrint G3 Mouse GE v2 8 × 60 K microarrays for mouse samples, or Agilent SurePrint G3 Rat GE v2 8 × 60 K microarrays for rat samples. Hybridization was performed for 17 h. Following hybridization, the slides were washed and scanned with Agilent Microarray Scanner (model G2505C)). Data were extracted using Agilent Feature Extraction software (version 12.0.2.2).

### 2.7. Transcriptomic Data Preprocessing

Transcriptomic data used in this study included previously published data by Poulsen et al. [7], where mice were exposed to NM-401 (available on NCBI GEO under the accession number GSE55286), along with newly generated data described herein.

Preprocessing of the newly generated two-color microarray data was performed using the R Shiny application eUTOPIA (https://github.com/Greco-Lab/eUTOPIA, accessed 13 September 2024) [40]. Mouse and rat data were treated as separate datasets. The mouse dataset was further divided into two: newly generated data (NM-403 exposure) and previously published NM-401 data (GSE55286), due to differences in experimental design and array configurations. Raw data and associated metadata files describing the associated biological and technical variables were uploaded to eUTOPIA. Quality control was performed using the arrayQualityMetrics package (version 3.52.0) [41]. Filtering methodology was based on the eUTOPIA tool and was applied consistently across datasets [40]. Outliers were identified and removed if flagged by at least two of the three quality metrics. Two mouse samples (NM-403) and five rat samples were excluded, leaving at least four samples per experimental group. Low-intensity probes were filtered by comparing intensity values to the negative control probes. Probes with intensities below the 90% quantile of the negative control probes in at least 80% of samples were excluded. Intensity values were log2-transformed and quantile-normalized across arrays. Technical variation (e.g., dye, array slide, position) was corrected using ComBat from the R package sva (version 3.44.0) [42]. Gene annotations were mapped to Ensembl gene IDs using platform-specific annotation files. When multiple probes mapped to the same gene, the median intensity was used.

Differential gene expression between exposure groups and corresponding controls was assessed using the limma method [43]. The model incorporated relevant batch covariates. Genes with absolute fold change >1.5 and Benjamini & Hochberg adjusted *p*-values < 0.05 were considered differentially expressed.

The previously published dataset (GSE55286) was treated as a one-color experiment due to its design, which analyzed only the Cy5 channel. Preprocessing followed the same quality control and normalization steps, with additional batch correction using surrogate variable analysis (SVA) to adjust for hidden confounders. Only genes present in both the GSE55286 and newly generated datasets were retained for downstream comparison.

### 2.8. Functional Enrichment

KEGG pathway enrichment analysis of the transcriptomics data was conducted using the R Shiny tool FunMappOne (https://github.com/Greco-Lab/FunMappOne, accessed 21 June 2023) [44]. Lists of DEGs were uploaded as input, and default background was selected for the analysis. Pathways with False Discovery Rate (FDR)-corrected *p*-value < 0.05 were considered significantly enriched. Enriched pathways were categorized into KEGG pathway groups based on the hierarchical organization provided by the FunMappOne tool and results for mouse and rat were matched by the pathway names into one plot. We chose KEGG pathway analysis rather than GO enrichment because KEGG provides a harmonized, cross-species framework, whereas differences in GO annotation depth between mouse and rat could confound the interpretation of species-specific effects.

## 3. Results

### 3.1. Neutrophil Influx

**Figure 1:**
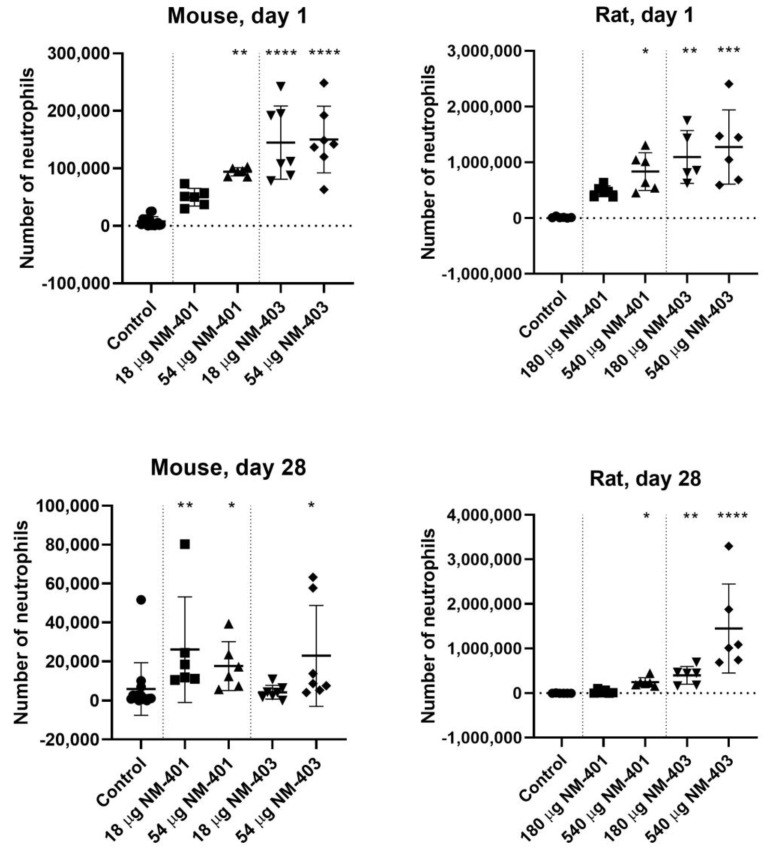
Number of neutrophils in bronchoalveolar lavage (BAL) fluid 1 and 28 days post-exposure in mice and rats. Data are shown as individual values, mean ± SD. Symbols: * *p* ≤ 0.05, ** *p* ≤ 0.01, *** *p* ≤ 0.001, **** *p* ≤ 0.0001 vs. control (Dunn’s method).

Both MWCNTs triggered a significant increase in neutrophils in BAL fluid in both species at 1 day post-exposure (Figure 1). On a mass-basis, NM-403 induced a stronger neutrophilic response than NM-401, consistent with its larger specific surface area. By day 28, neutrophil levels had declined but remained elevated in both mice and rats, with a more persistent response observed in rats.

**Figure 2:**
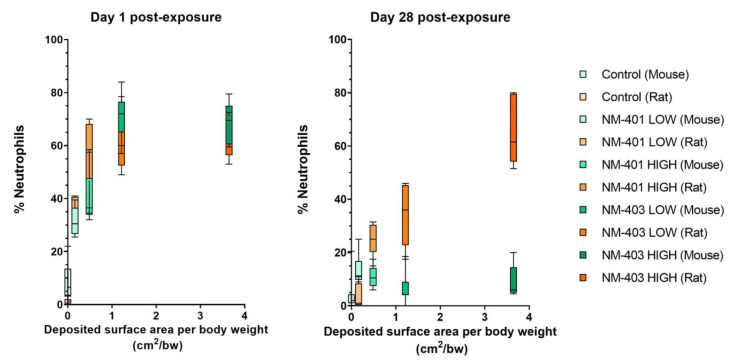
Percentage of neutrophils in bronchoalveolar lavage (BAL) fluid relative to deposited surface area per body weight (cm^2^/g bw) (for NM-401 and NM-403 in mice and rats at 1 and 28 days post-exposure.

When normalized to deposited surface area per kg bw (Figure 2), the neutrophil percentage in BAL fluid showed a similar dose–response curve in both species at 1 day. However, at day 28, rats displayed a more robust inflammatory response, and especially NM-403 elicited greater neutrophilic influx in rats as compared to mice.

### 3.2. Transcriptomic Alterations

**Figure 3:**
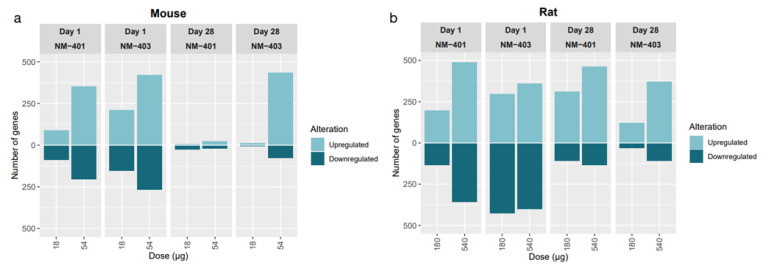
Bar plots showing upregulated and downregulated DEGs in mouse (**a**) and rat (**b**) lungs at days 1 and 28 post-exposure to NM-401 and NM-403. Light-blue bars indicate upregulated genes, while dark-blue bars represent downregulated genes.

**Figure 4:**
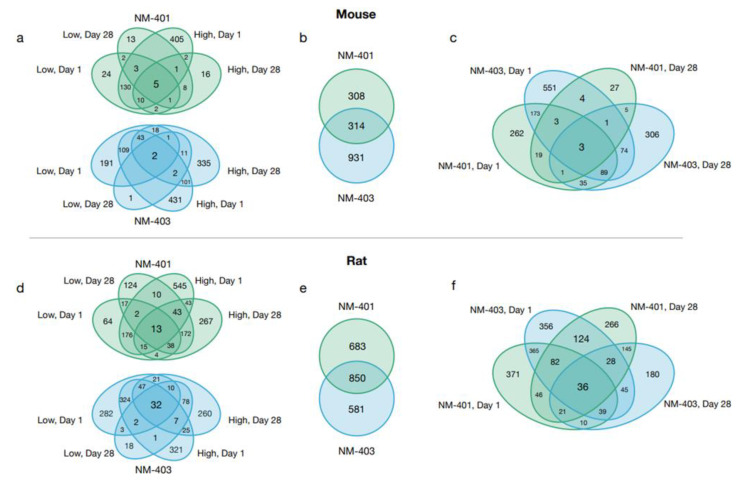
Venn diagrams illustrating DEG overlaps in mice (**a**–**c**) and rats (**d**–**f**). Blue = NM-403, Green = NM-401. Panels (**a**,**d**) display overlaps of low and high exposure levels at both time points for each MWCNT individually. Panels (**b**,**e**) show the overall DEG overlap between the two MWCNTs. Panels (**c**,**f**) highlight overlaps among MWCNT-time point pairs.

Initial analysis of transcriptomic alterations revealed distinct sets of differentially expressed genes (DEGs) per exposure condition (Figure 3). DEG counts increased dose-dependently for both NM-401 and NM-403 in both species, though patterns and magnitude of responses differed. In mice, day 1 responses—especially at high doses—showed the greatest transcriptomic changes with NM-403 producing more DEGs, largely due to strong upregulation. By day 28, responses diminished except for high-dose NM-403. In rats, transcriptomic alterations were also strongest at day 1, but substantial DEG counts persisted through day 28, indicating a prolonged response in parallel with the stronger neutrophil influx observed in rats on day 28. Venn Diagrams (Figure 4) illustrate DEG overlaps across doses and time points for each MWCNT and species.

In both species, NM-401 and NM-403 triggered substantial transcriptional changes in the lung, with some shared DEGs across doses and time points. For NM-401 in mice, five genes were consistently differentially expressed across all exposure conditions, while NM-403 had two such genes. In rats, the overlap was more prominent, with 13 shared DEGs across NM-401 conditions and 32 across NM-403 conditions. This suggests that, in both species, some gene responses are conserved regardless of dose or time, especially in rats.

When comparing NM-401 and NM-403 within each species, there was a significant overlap in DEGs. In mice, 314 genes were shared between the two materials, and in rats, 850 genes were shared, indicating that both materials elicit broadly similar transcriptional responses in the lung. Additionally, cross-material and cross-time comparisons showed overlapping DEGs in both species—although to a greater extent in rats—with 3 genes shared in all mouse groups and 36 in all rat groups. Overall, while there are some condition- and species-specific differences, the data show that NM-401 and NM-403 induce partially overlapping lung transcriptional responses.

In mice, the only gene common across all exposure groups was C-C motif chemokine ligand 17 (*Ccl17*), which was present in all groups except the low dose of NM-401 at 28 day post-exposure (Supplementary Figure S1B). In rats, six DEGs were common across all conditions: resistin like alpha (*Retnla*), matrix metallopeptidase 12 (*Mmp12*), lipocalin 2 (*Lcn2*), complement component 3 (*C3*), solute carrier family 26, member 4 (*Slc26a4*) and LOC102551003 (codes for keratin associated protein 20-2-like 1 (*Krtap20-2l1*)) (Supplementary Figure S1A).

Due to differences in gene annotation and hybridization platforms, direct gene-level comparison between species was not performed. Instead, DEGs were compared using previously published fibrosis gene signatures [6,45] and acute phase response genes linked to cardiovascular disease [24].

### 3.3. Fibrosis

We assessed fibrosis-related gene expression using two established transcriptional panels: the 24-gene fibrosis signature from Snyder-Talkington et al. [45], and the 17-gene panel from Rahman et al. [46]. Although these panels were established using murine data, all genes were confirmed to be expressed in rats.

Overall, the number of significantly regulated fibrosis-related genes was somewhat higher in rats than in mice across time points and exposure groups. In rats, up to 18 fibrosis-related genes were differentially expressed, while in mice the maximum was 19. This corresponds to expression of up to 79% of the Snyder-Talkington genes and up to 100% of the Rahman genes expressed under certain conditions, highlighting the cross-species utility of these transcriptional markers in detecting nanomaterial-induced fibrotic responses.

NM-403 consistently induced stronger transcriptional responses than NM-401, especially at the high dose and at day 28. This was most evident in rats, where NM-403 exposure resulted in a higher number of DEGs and greater log fold changes (logFC) for overlapping fibrosis-related genes at both dose levels. Additionally, gene expression exhibited dose-dependency for several key genes, with highest expression levels typically seen at day 28 in high-dose groups.

Among the most upregulated genes in mice were tissue inhibitor of TIMP metallopeptidase inhibitor 1 (*Timp1*), FXYD domain containing ion transport regulator 4 (*Fxyd4*), C-C motif chemokine ligand 2 (*Ccl2*), *Ccl17*, and arginase 1 (*Arg1*), with *Timp1* reaching a logFC of 3.50 at day 28 following high-dose NM-403. In rats, even greater fold changes were observed, with *C3*, *Ccl2*, *Timp1*, *Slc26a4*, and C-type lectin domain family 4 member A (*Clec4a2*) among the top responders. Notably, *C3* showed a logFC of 5.58 and *Timp1* a logFC of 5.25, indicating a potent fibrotic transcriptional response.

Across both species, a core set of genes—*Timp1*, *Ccl2*, *Ccl17*, *C3*, and *Arg1*—was consistently upregulated. These genes are functionally associated with extracellular matrix remodeling (*Timp1*), chemotaxis (*Ccl2*, *Ccl17*), and immune activation (*C3*, *Arg1*) [6,46], underscoring their role in early fibrotic processes across rodent models in response to MWCNTs (Table 4 and Table 5).

### 3.4. Acute Phase Response

We examined acute phase response genes identified via NCBI (https://www.ncbi.nlm.nih.gov/), finding 61 different genes in mice and 47 in rats. The number and identity of differentially expressed acute phase response genes are summarized in Table 6 and Table 7, with more genes observed at higher NM doses.

Several genes, including *Lcn2*, orosomucoid 1 (*Orm1*), haptoglobin (*Hp*), interleukin 1 receptor antagonist (*Il1rn*), and inter alpha-trypsin inhibitor, heavy chain 4 (*Itih4*), were commonly regulated in both species. Among the shared genes, *Lcn2* and *Orm1* exhibited consistent and dose-dependent upregulation in both species, making them strong candidates as general biomarkers of NM-induced acute phase responses. In rats, *Lcn2* expression remained elevated through day 28, reflecting a prolonged systemic response, while mice showed peak expression primarily at high doses or later time points. Mouse-specific genes *Saa3* and *Orm2* showed sharp upregulation at day 1 (logFC = 5.5 and 2.5 for *Saa3* and *Orm2*, respectively) followed by partial reduction by day 28, though expression remained elevated at the highest dose.

**Figure 5:**
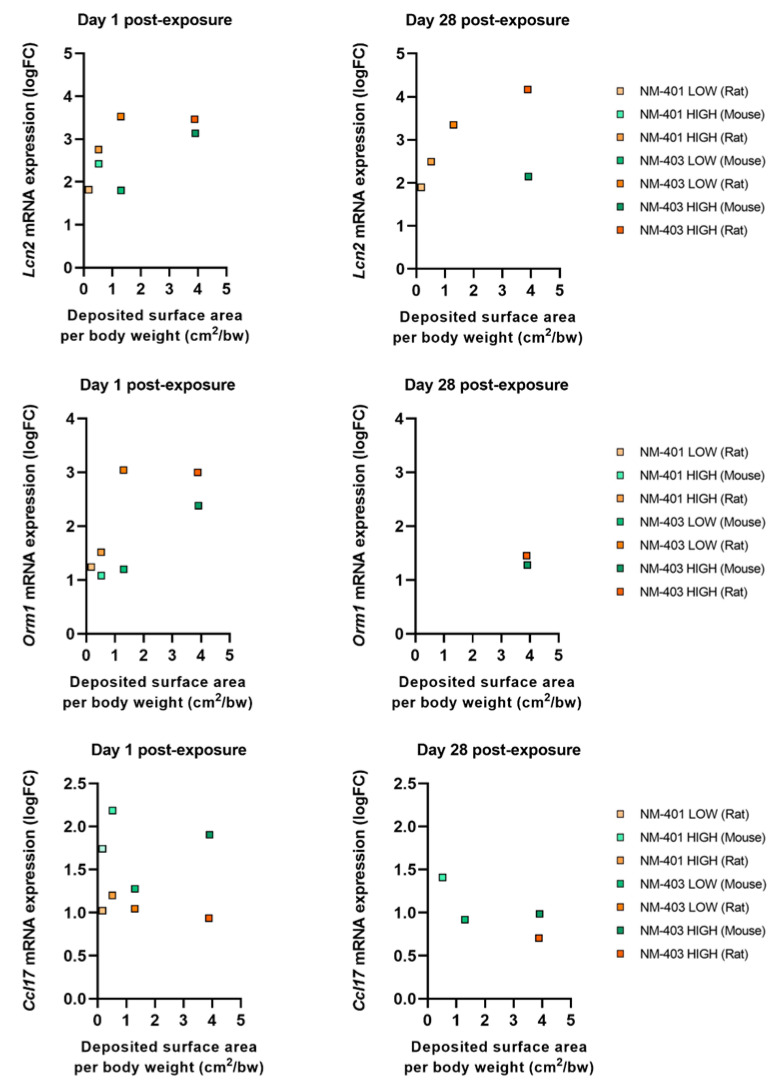
Fold change in *Lcn2*, *Orm1*, and *Ccl17* expression in the lungs of mice and rats as a function of deposited surface area per body weight (cm^2^/g bw).

**Figure 6:**
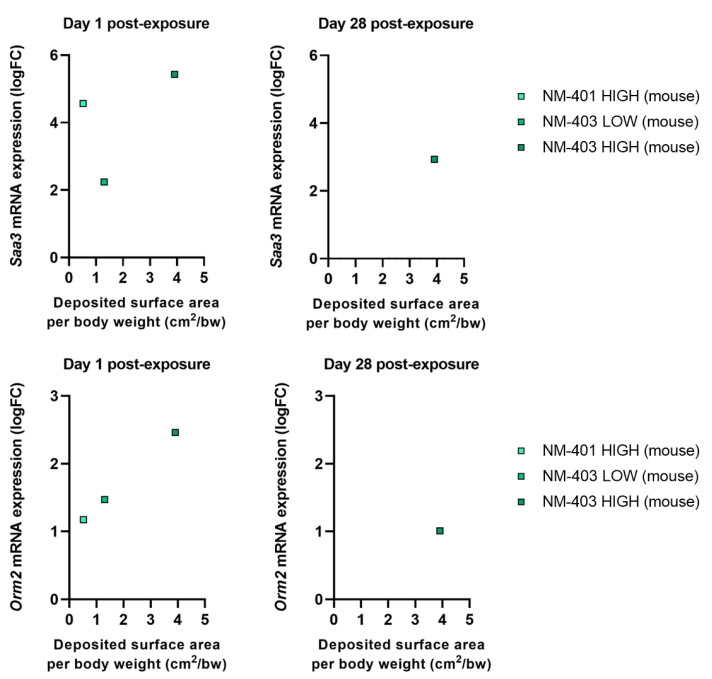
Fold change in *Saa3* and *Orm2* expression in the lungs of mice as a function of the deposited surface area per body weight (cm^2^/g bw).

When dose was normalized to deposited surface area, both *Lcn2* and *Orm1* continued to show robust dose-dependent expression in both species and at both time points (Figure 5). *Lcn2*, in particular, demonstrated persistent induction through day 28, especially in rats, emphasizing its role in sustained acute phase response. *Orm1* showed a similar but slightly more attenuated trend. The chemokine *Ccl17* was modestly upregulated on day 1 in both species but declined by day 28, indicating a transient role in early immune cell recruitment (Figure 5). In mice, *Saa3* and *Orm2* expression patterns (Figure 6) confirmed their early, dose-dependent induction and partial resolution over time. Overall, the acute phase response was more persistent in rats than in mice, aligning with prolonged neutrophilic inflammation and fibrosis-associated gene expression in the rat lung.

### 3.5. Functional Enrichment

We conducted KEGG pathway enrichment analyses to compare functional transcriptomic responses between mice and rats. While the broader transcriptomic effects of MWCNT exposure have been extensively described [19,21,47,48,49,50,51,52,53], this section emphasizes interspecies comparison.

**Figure 7:**
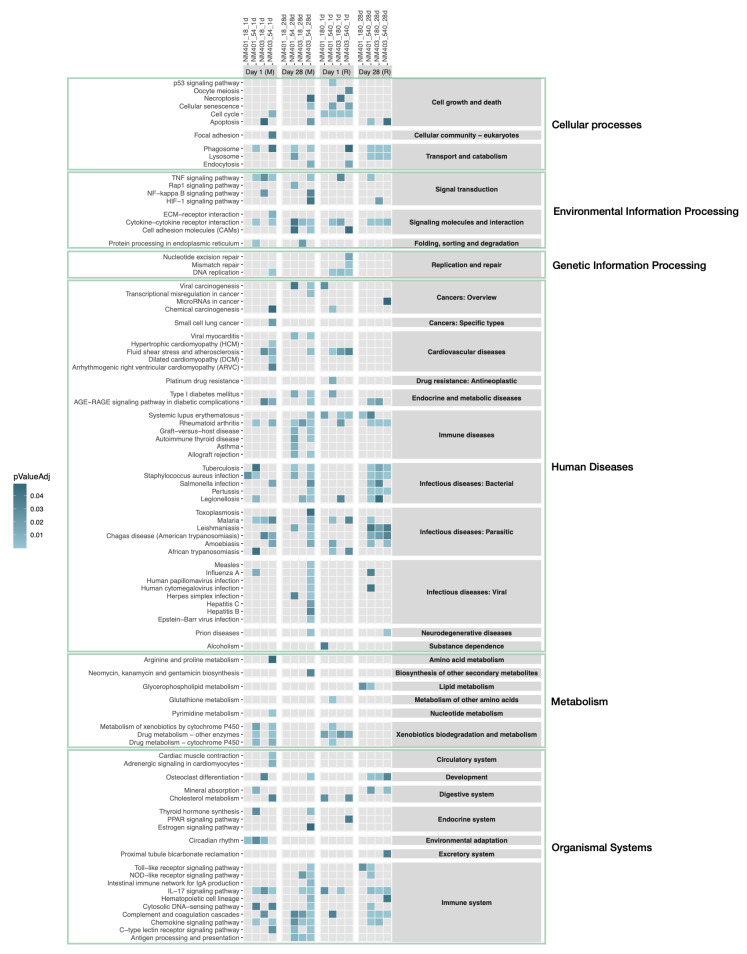
KEGG pathway enrichment by experiment. Significant enrichment is indicated by blue cells in the heatmap. The intensity of the blue color corresponds to the FDR-adjusted *p*-value of the enrichment, with lighter blue indicating a lower *p*-value. Light grey cells indicate no significant enrichment. Experiments are grouped by species (M for mouse, R for rat) and time points. KEGG pathways are organized by their categories at level 2 (grey boxes on the right side) and further categorized by level 1 (green rectangles).

Enrichment analysis of DEGs by experimental group revealed a dose-dependent increase in the number of enriched pathways. Higher doses of both NM-401 and NM-403 at both led to a greater number of KEGG-enriched pathways in both species (Figure 7, Supplementary Table S1).

Overall, NM-403 induced more enriched pathways than NM-401, particularly in mice. Interestingly, in mice exposed to NM-403, enrichment was similar between low and high doses, suggesting a possible saturation effect or heightened sensitivity at lower doses. In total, 78 pathways were significantly enriched in mice and 56 in rats, with 45 shared between the species in at least one condition (Supplementary Table S1), indicating considerable overlap despite species differences.

Key immune-related pathways—such as ‘Complement and coagulation cascades’, ‘Cytokine–cytokine receptor interaction’, ‘IL-17 signaling pathway’, ‘Phagosome’, and ‘Rheumatoid arthritis’—were consistently enriched across both species, time points, and doses, highlighting a conserved inflammatory and immune activation profile (Figure 7).

At a broader functional level, enriched pathways spanned 26 KEGG categories in mice and 23 in rats (out of 31 total categories). Eighteen of these categories were shared, including major biological functions such as ‘Xenobiotics biodegradation and metabolism’, ‘Replication and repair’, ‘Signal transduction’, ‘Signaling molecules and interaction’, ‘Transport and catabolism’, ‘Cell growth and death’, and multiple disease-associated categories like ‘Immune system’, ‘Endocrine and metabolic diseases’, ‘Cardiovascular diseases’, and ‘Infectious diseases’ (covering bacterial, viral, and parasitic infections).

Quantitatively, 45 of 78 pathways in mice (58%) and 18 of 26 KEGG categories (69%) were also observed in rats. Conversely, 45 of 56 pathways (80%) and 18 of 23 categories (78%) in rats overlapped with mouse results. Species-specific enrichments included 33 unique pathways in mice and 11 in rats. Most of these (22/33 in mice, 10/11 in rats) were enriched under a single experimental condition. In mice, three pathways—‘Circadian rhythm’, ‘C-type lectin receptor signaling pathway’, and ‘Antigen processing and presentation’—were enriched in three different conditions, all belonging to the ‘Immune system’ super-category. In rats, ‘Lipid metabolism’ was the only pathway enriched under multiple conditions.

## 4. Discussion

This study compared the pulmonary transcriptional responses of rats and mice following intratracheal instillation of two MWCNTs with distinct physicochemical properties: NM-401 a long, thick, and needle-like fiber, and NM-403, a short and thin fiber not meeting the FP criteria. Our aim was to assess to what extent rodent models exhibit concordant biological responses to nanomaterial exposure, using both traditional markers and transcriptomic signatures.

We used intratracheal instillation to ensure precise, dose-controlled delivery to the lungs [54]. Though inhalation is the gold standard for risk assessment, previous studies have demonstrated that intratracheal instillation effectively achieves widespread lung distribution [8,55,56]. When inhalation exposure is used, particle-specific differences in the size of the aerosolized particles or particle aggregates may greatly influence the pulmonary deposition and distribution between materials [15,22], and consequently, lung burden has to be determined experimentally to ensure comparability. Our prior work has demonstrated that rats exposed to matched doses of MWCNTs via intratracheal instillation or inhalation induces similar inflammatory responses [22]. This makes it a suitable for comparative hazard evaluation.

Neutrophilic inflammation was consistently observed in both species, with a clear dose-dependent increase at day 1 post-exposure to either MWCNTs. Notably, the magnitude of this acute response was greater for NM-403, which has a larger surface area, suggesting that particle surface area is an important determinant of pulmonary interaction and inflammatory potency. However, the apparent dose–response relationship with dosed surface area was attenuated at the highest surface dose at day 1 (Figure 2). This attenuation most likely reflects the limited dynamic range of the chosen outcome measure (% neutrophils), which becomes less sensitive at high levels of inflammation. We used % neutrophils in order to enable direct comparison between mice and rats, since the absolute numbers of BAL cells differ vastly between the species.

Despite species-specific differences in the persistence of inflammation—rats showing a more prolonged response—both rats and mice retained elevated neutrophil levels at day 28, indicating a shared pattern of sustained inflammatory signaling. When doses were normalized to deposited surface area, the early-phase dose–response relationships were highly similar across species.

Transcriptomic profiling further revealed strong concordance in gene expression responses between species and materials. A dose-dependent trend in DEGs was observed for both NM-401 and NM-403 in rats and mice. In both species, transcriptomic responses peaked at day 1 post-exposure and were especially pronounced at higher doses, correlating with neutrophilic influx. While mice generally showed a transient gene expression profile, with diminished responses by day 28, rats retained substantial DEG counts at this later time point—mirroring the prolonged inflammation. Despite these temporal differences, substantial overlaps in DEGs were detected across time points, doses, and materials within each species. Importantly, hundreds of DEGs were shared between NM-401 and NM-403 exposures in both rats and mice, suggesting broadly similar molecular responses regardless of MWCNT structure. A smaller but notable set of DEGs was consistently regulated across all exposure conditions within each species, highlighting conserved transcriptional responses to MWCNTs. The shared transcriptional responses to CNTs with different physical dimensions have also been reported earlier [7,19,21,51,57].

Previous studies have demonstrated that inhalation of both long, thick Mitsui-7 MWCNT [3] and short, thin MWCNTs can induce fibrosis in rats [2]. In the context of intratracheal instillation, earlier research has shown that NM-401 induces fibrosis in both rats and mice, whereas NM-403 does not. In rats, fibrosis was observed 28 days after NM-401 exposure at the high dose (540 µg/rat), while NM-403 only caused minor interstitial thickening without fibrosis [22]. In mice, focal fibrosis was observed only at a higher dose (162 µg/mouse) [7], whereas no fibrosis was detected one year after lower-dose exposure (54 µg/mouse) [32].

To further explore potential fibrogenic mechanisms, we analyzed transcriptomic changes using established fibrosis gene panels [45,46]. Here again, both rats and mice showed dose-dependent upregulation of key fibrosis-related genes following exposure to both MWCNTs. Rats exhibited a greater number and magnitude of fibrosis-related DEGs—particularly at day 28 and following high-dose NM-403 exposure—indicating a more sustained fibrotic response compared to mice. Importantly, a conserved set of fibrosis-related genes, including *Timp1*, *Ccl2*, *Ccl17*, *C3*, and *Arg1*, was upregulated across both species, highlighting common pathways of fibrotic signaling.

Inflammation is a critical driver of fibrosis [17]. Our findings indicate that neutrophilic inflammation and profibrotic gene expression are closely interconnected in MWCNT-induced lung toxicity. The *C3* up-regulation suggest early complement activation leading to rapid neutrophil recruitment. Progression to C5 cleavage and C5a–C5aR1 signaling likely amplifies neutrophilic influx, macrophage activation, and profibrotic signaling [58]. In parallel, IL-4/STAT6-dependent macrophage polarization (*Arg1*, *Timp1*) establishes a tissue-remodeling milieu, supporting extracellular matrix deposition [59]. Chemokine circuits such as Ccl2 and Cxcl family members further link neutrophil influx to monocyte/macrophage recruitment, underlining cross-species concordance of conserved fibrotic pathways.

Interestingly, NM-403 induced stronger transcriptomic responses than NM-401 in many conditions, especially at high doses. This was reflected not only in the number of DEGs and fibrosis-associated genes but also in the expression magnitude of acute phase and inflammation-related markers. These findings suggest that surface area contribute to enhanced biological reactivity. Differences in physical properties between NM-401 and NM-403 (e.g., fiber length and rigidity) may influence the relative contribution of complement activation and macrophage polarization, which could help explain the stronger late neutrophilic and fibrotic responses observed with NM-403.

The acute phase response is a dominant transcriptional signature following pulmonary exposure to nanomaterials in mice [24,60,61] including MWCNTs [9,21]. Importantly, the acute phase protein SAA is implicated in atherosclerosis [26], and acute phase proteins have been linked to future cardiovascular disease risk in epidemiological studies [57,62,63,64]. Previous research has demonstrated a dose–response relationship in nanomaterial-induced acute phase response, both in the number of differentially expressed genes and the magnitude of gene induction [24,65].

Analysis of the acute phase response revealed another layer of similarity between rats and mice. Shared upregulation of genes such as *Orm1*, *Lcn2*, *Hp*, and *Il1rn* was observed in both species, consistent with systemic inflammation. *Lcn2* in particular showed persistent and robust expression in rats, and to a lesser degree in mice, highlighting its potential as a cross-species biomarker of nanomaterial-induced systemic effects. While mice also exhibited strong upregulation of *Saa3* and *Orm2*, these genes are not expressed in rats, indicating divergence in acute phase signaling.

Direct comparison of gene expression levels between species presents inherent challenges due to differences in microarray platforms, gene annotations, and underlying biology. Although mice and rats share high genomic homology, gene-level analyses are complicated by species-specific expression patterns and platform-dependent variability. To address these limitations, this study focused on cross-species comparison at the functional level using the KEGG database, which provides curated pathways for both species [66,67]. Although the KEGG dataset for mice is more extensive than that for rats, all 78 KEGG pathways enriched in mice were also available in the database for rats.

The analyses demonstrated that both species exhibited a dose-dependent increase in the number of enriched pathways, with NM-403 consistently inducing more enriched pathways than NM-401. Importantly, 45 of the enriched pathways were shared between species, including key immune-related pathways such as ‘Cytokine–cytokine receptor interaction,’ ‘IL-17 signaling pathway,’ and ‘Phagosome formation.’ These overlaps indicate that fundamental immune and inflammatory processes are commonly activated in response to MWCNTs in both species.

At a broader level, mice and rats shared 18 of 31 KEGG functional categories, and enrichment patterns included multiple pathways related to immune signaling, metabolism, xenobiotic detoxification, and infectious disease responses. This points to a conserved transcriptional architecture in response to MWCNTs.

Despite several shared immune and inflammatory pathways enriched in both species, a subset of pathways was uniquely enriched in either mice or rats, indicating species-specific aspects of the response to MWCNT exposure. These differences likely reflect underlying biological divergence and variability in how each species processes and responds to MWCNTs. In rats, uniquely enriched pathways were primarily associated with genetic information processing and repair, particularly at day 1, suggesting a more pronounced early response involving cell cycle regulation and DNA damage repair. In contrast, mice showed enrichment of pathways related to viral infection responses, most notably at the high dose of NM-403 at day 28, potentially indicating broader activation of antiviral or interferon-related signaling cascades. At day 28, the antigen processing and presentation pathway was also enriched in mice, which could suggest sustained or adaptive immune engagement at later time points.

Moreover, the data revealed considerable variability across exposure groups within species, with several pathways enriched in only a single condition. This suggests that within-species variability across exposure types and time points may exceed the overall differences observed between species. Collectively, these findings underscore that while key innate immune processes are conserved, species-specific modulation of cell cycle regulation, immune processing, and pathogen response pathways adds a layer of complexity to the interpretation and cross-species comparisons in nanomaterial toxicity.

Our results show sustained inflammatory and fibrotic responses to both long NM-401 and short NM-403 across species. While IARC and ECHA have classified FP-compatible MWCNTs as carcinogenic [29,33], studies also show that non-fiber-compatible forms can elicit persistent lung pathology including fibrosis [2] and cancer [10,11]. Thus, both categories converge on inflammation-driven AOPs and remain relevant for regulatory considerations [30]. Importantly, non-FP-compatible MWCNTs such as NM-403 are not currently covered by the adopted regulatory classifications, underscoring the need for further long-term studies to fully evaluate their pathogenic potential and to determine whether the fiber paradigm should be extended to encompass such materials.

## 5. Conclusions

This study demonstrates a high degree of concordance between rats and mice in terms of inflammation, transcriptomic alterations, fibrosis-associated signaling, and acute phase responses following MWCNT exposure. NM-403 generally elicited stronger responses than NM-401, particularly at high doses, likely due to its larger specific surface area. Importantly, our findings show that even short, non-fiber-like MWCNTs can provoke potent and persistent pulmonary effects, challenging assumptions based solely on MWCNT properties. Despite some differences in the magnitude and duration of responses, the core biological pathways activated were highly conserved between species. These results underscore the relevance of both rats and mice as complementary models for evaluating the pulmonary hazards of high-aspect-ratio nanomaterials.

## Figures and Tables

**Table 1:** MWCNT properties.

	Diameter (nm)	Length (nm)	Specific Surface Area (m^2^/g)	Purity (%)	Metal Content (wt%)
**NM-401**	67 (±24)	4048 (±2371)	18	98	Fe (0.05) Mg (0.015)
**NM-403**	12 (±7)	443 (±222)	135	96.6	Fe (0.002) Mg (0.188) Co (1.2) Ni (0.0018) Mn (0.16)

**Table 2:** MWCNT doses expressed in mg/kg bw.

Dose (Mass)	Body Weight (bw)	Dose (Mass) mg/kg bw
18 µg (mouse)	18.64 g	0.966
54 µg (mouse)	18.64 g	2.897
180 µg (rat)	187.7 g	0.959
540 µg (rat)	187.7 g	2.877

**Table 3:** MWCNT doses in terms of total deposited surface area.

Dose (Mass) in Mice	Dosed Surface Area (cm^2^)	Deposited Surface Area (cm^2^/g bw)
18 µg NM-401	0.018 mg × 180 cm^2^/mg = 3.24	3.24 cm^2^/18.64 g = 0.174
54 µg NM-401	0.054 mg × 180 cm^2^/mg = 9.72	9.72 cm^2^/18.64 g = 0.522
18 µg NM-403	0.018 mg × 1350 cm^2^/mg = 24.3	24.3 cm^2^/18.64 g = 1.304
54 µg NM-403	0.054 mg × 1350 cm^2^/mg = 72.9	72.9 cm^2^/18.64 g = 3.911
**Dose (Mass) in Rats**		
180 µg NM-401	0.18 mg × 180 cm^2^/mg = 32.4	32.4 cm^2^/187.7 g = 0.173
540 µg NM-401	0.54 mg × 180 cm^2^/mg = 97.2	97.2 cm^2^/187.7 g = 0.518
180 µg NM-403	0.18 mg × 1350 cm^2^/mg = 243	243 cm^2^/187.7 g = 1.294
540 µg NM-403	0.54 mg × 1350 cm^2^/mg = 729	729 cm^2^/187.7 g = 3.884

**Table 4:** The number of differentially expressed fibrosis-related genes, based on Snyder-Talkington et al. (genes shown in **bold**) or Rahman et al., in lung tissue from mice exposed to NM-401 or NM-403. Values represent log fold changes (LogFC).

Day 1								Day 28							
NM-401				NM-403				NM-401				NM-403			
Low Dose		High Dose		Low Dose		High Dose		Low Dose		High Dose		Low Dose		High Dose	
Gene	LogFC	Gene	LogFC	Gene	LogFC	Gene	LogFC	Gene	LogFC	Gene	LogFC	Gene	LogFC	Gene	LogFC
		***Adora***	(1.41)												
		***Adora2b***	(0.62)												
		*Arg1*	(2.38)			*Arg1*	(1.33)								
														*C1qb*	(1.43)
***C3***	(0.82)	***C3***	(1.21)	***C3***	(1.12)	***C3***	(1.80)							***C3***	(1.11)
		*Ccl9*	(1.29)			*Ccl9*	(0.95)							*Ccl9*	(1.22)
***Ccl17***	(1.74)	***Ccl17***	(2.19)	***Ccl17***	(1.28)	***Ccl17***	(1.90)			***Ccl17***	(1.41)	***Ccl17***	(0.92)	***Ccl17***	(0.99)
		***Ccl2***	(1.84)			***Ccl2***	(2.30)							***Ccl2***	(2.32)
*Ccr5*	(0.80)	*Ccr5*	(1.12)											*Ccr5*	(0.96)
														*Ctss*	(1.08)
		*Fcgr2b*	(1.27)			*Fcgr2b*	(0.69)							*Fcgr2b*	(0.67)
*Fxyd4*	(2.72)	*Fxyd4*	(3.53)			*Fxyd4*	(1.53)							*Fxyd4*	(1.11)
		***Il1b***	(1.04)			***Il1b***	(1.38)							***Il1b***	(0.69)
						***Il1rn***	(0.80)							***Il1rn***	(1.31)
		***Il6***	(1.54)			***Il6***	(1.69)							***Il6***	(1.28)
														***Irf7***	(2.10)
														*Ly86*	(0.64)
				***Mmp12***	(0.83)	***Mmp12***	(1.90)							***Mmp12***	(2.35)
		*S100a4*	(0.66)												
		***Selp***	(0.95)												
*Serpina3n*	(1.07)	*Serpina3n*	(1.62)	*Serpina3n*	(0.85)	*Serpina3n*	(1.97)							*Serpina3n*	(0.72)
*Slc26a4*	(3.50)	*Slc26a4*	(4.36)	*Slc26a4*	(1.06)	*Slc26a4*	(3.09)							*Slc26a4*	(3.08)
***Socs1***	(0.79)	***Socs1***	(1.10)	***Socs1***	(−0.61)										
***Timp1***	(1.65)	***Timp1***	(3.08)	***Timp1***	(1.28)	***Timp1***	(3.50)							***Timp1***	(1.36)
														***Tnfaip3***	(0.80)
**8 genes**		**18 genes**		**7 genes**		**14 genes**		**0 genes**		**1 gene**		**1 gene**		**19 genes**	

**Table 5:** The number of differentially expressed fibrosis-related genes, based on Snyder-Talkington et al. (genes shown in **bold**) or Rahman et al., in lung tissue from rats exposed to NM-401 or NM-403. Values represent log fold changes (LogFC).

Day 1								Day 28							
NM-401				NM-403				NM-401				NM-403			
Low Dose		High Dose		Low Dose		High Dose		Low Dose		High Dose		Low Dose		High Dose	
Gene	LogFC	Gene	LogFC	Gene	LogFC	Gene	LogFC	Gene	LogFC	Gene	LogFC	Gene	LogFC	Gene	LogFC
														***Adora2b***	(1.04)
		*Arg1*	(0.76)							*Arg1*	(0.82)	*Arg1*	(1.09)	*Arg1*	(2.00)
										*C1qb*	(0.67)			*C1qb*	(0.72)
***C3***	(1.55)	***C3***	(1.80)	***C3***	(1.54)	***C3***	(5.58)	***C3***	(1.21)	***C3***	(1.66)	***C3***	(2.21)	***C3***	(2.29)
								*Ccl9*	(1.71)	*Ccl9*	(2.84)	*Ccl9*	(1.70)	*Ccl9*	(1.78)
***Ccl17***	(1.02)	***Ccl17***	(1.20)	***Ccl17***	(1.05)	***Ccl17***	(3.29)							***Ccl17***	(0.71)
***Ccl2***	(1.16)	***Ccl2***	(2.77)	***Ccl2***	(1.63)	***Ccl2***	(5.26)			***Ccl2***	(1.96)	***Ccl2***	(1.71)	***Ccl2***	(2.13)
		*Ccr5*	(0.83)			*Ccr5*	(3.75)								
		*Ch25h*	(1.01)					*Ch25h*	(1.05)	*Ch25h*	(1.31)			*Ch25h*	(1.23)
		*Cle4a2*	(0.64)	*Clec4a2*	(0.75)	*Clec4a2*	(4.10)								
										*Ctss*	(0.70)	*Ctss*	(0.64)	*Ctss*	(0.72)
		*Fcgr2b*	(0.74)			*Fcgr2b*	(1.13)			*Fcgr2b*	(0.86)			*Fcgr2b*	(1.12)
		***Il1b***	(0.76)	***Il1b***	(1.03)					***Il1b***	(0.94)				
		***Il1r1***	(0.90)												
		***Il1rn***	(1.26)	***Il1rn***	(0.72)					***Il1rn***	(1.40)	***Il1rn***	(1.03)	***Il1rn***	(1.12)
										*Itgb2*	(0.77)				
***Mmp12***	(1.33)	***Mmp12***	(1.60)	***Mmp12***	(1.28)	***Mmp12***	(3.89)	***Mmp12***	(1.07)	***Mmp12***	(3.02)	***Mmp12***	(1.87)	***Mmp12***	(2.47)
*Retnla*	(2.71)	*Retnla*	(2.63)	*Retnla*	(1.53)	*Retnla*	(3.58)	*Retnla*	(1.65)	*Retnla*	(2.47)	*Rentla*	(1.92)	*Rentla*	(2.75)
		***Sele***	(0.80)												
*Serpina3n*	(0.94)	*Serpina3n*	(1.93)	*Serpina3n*	(−0.79)	*Serpina3n*	(−0.75)			*Serpina3n*	(0.91)				
*Slc26a4*	(0.89)	*Slc26a4*	(1.47)	*Slc26a4*	(1.17)	*Slc26a4*	(4.34)	*Slc26a4*	(1.26)	*Slc26a4*	(2.16)	*Slc26a4*	(1.96)	*Slc26a4*	(2.88)
***Timp1***	(0.83)	***Timp1***	(1.47)	***Timp1***	(0.61)	***Timp1***	(5.25)			***Timp1***	(0.60)				
		***Tnf***	(1.24)	***Tnf***	(0.76)			***Tnf***	(1.11)	***Tnf***	(1.45)				
**8 genes**		**18 genes**		**12 genes**		**11 genes**		**7 genes**		**17 gene**		**9 gene**		**14 genes**	

**Table 6:** Differentially expressed acute phase response genes in mice, based on a total of 61 identified genes. Values represent log fold changes (LogFC).

Day 1								Day 28							
NM-401				NM-403				NM-401				NM-403			
Low Dose		High Dose		Low Dose		High Dose		Low Dose		High Dose		Low Dose		High Dose	
Gene	LogFC	Gene	LogFC	Gene	LogFC	Gene	LogFC	Gene	LogFC	Gene	LogFC	Gene	LogFC	Gene	LogFC
				*Angptl4*	(1.56)	*Angptl4*	(1.83)								
						*Fn1*	(0.91)								
						*Hamp*	(−1.06)								
						*Hp*	(0.77)								
		*Il1b*	(1.04)			*Il1b*	(1.38)							*Il1b*	(0.69)
						*Il1rn*	(0.80)							*Il1rn*	(1.31)
		*Il6*	(1.54)			*Il6*	(1.69)							*Il6*	(1.28)
*Itih4*	(1.06)	*Itih4*	(1.09)	*Itih4*	(1.40)	*Itih4*	(1.63)							*Itih4*	(1.60)
		*Lcn2*	(2.42)	*Lcn2*	(1.80)	*Lcn2*	(3.14)							*Lcn2*	(2.15)
		*Orm1*	(1.08)	*Orm1*	(1.20)	*Orm1*	(2.38)							*Orm1*	(1.28)
		*Orm2*	(1.17)	*Orm2*	(1.47)	*Orm2*	(2.46)							*Orm2*	(1.01)
		*Reg3g*	(2.82)												
		*Selp*	(0.95)												
*Serpina3n*	(1.07)	*Serpina3n*	(1.62)	*Serpina3n*	(0.85)	*Serpina3n*	(1.97)							*Serpina3n*	(0.72)
		*Saa1*	(3.17)			*Saa1*	(4.45)							*Saa1*	(1.20)
		*Saa2*	(1.77)	*Saa2*	(1.50)	*Saa2*	(3.48)								
		*Saa3*	(4.57)	*Saa3*	(2.24)	*Saa3*	(5.43)							*Saa3*	(2.93)
**2 genes**		**12 genes**		**8 genes**		**15 genes**		**0 genes**		**0 genes**		**0 genes**		**10 genes**	

**Table 7:** Differentially expressed acute phase response genes in rats, based on a total of 47 identified genes. Values represent log fold changes (LogFC).

Day 1								Day 28							
NM-401				NM-403				NM-401				NM-403			
Low Dose		High Dose		Low Dose		High Dose		Low Dose		High Dose		Low Dose		High Dose	
Gene	LogFC	Gene	LogFC	Gene	LogFC	Gene	LogFC	Gene	LogFC	Gene	LogFC	Gene	LogFC	Gene	LogFC
		*Fn1*	(1.06)							*Fn1*	(0.67)				
				*Hp*	(0.78)			*Hp*	(0.84)	*Hp*	(0.78)	*Hp*	(1.49)	*Hp*	(1.80)
		*Il1rn*	(1.23)	*Il1rn*	(0.72)					*Il1rn*	(1.40)	*Il1rn*	(1.03)	*Il1rn*	(1.12)
						*Itih4*	(0.61)							*Itih4*	(0.76)
						*Kng1*	(0.69)					*Kng1*	(1.16)	*Kng1*	(1.54)
												*Lbp*	(0.75)	*Lbp*	(1.03)
*Lcn2*	(1.82)	*Lcn2*	(2.79)	*Lcn2*	(3.53)	*Lcn2*	(3.46)	*Lcn2*	(1.90)	*Lcn2*	(2.50)	*Lcn2*	(3.35)	*Lcn2*	(4.17)
*Orm1*	(1.24)	*Orm1*	(1.52)	*Orm1*	(0.76)	*Orm1*	(3.00)							*Orm1*	(1.45)
*Plscr1*	(−0.61)	*Plscr1*	(−0.74)			*Plscr1*	(−0.88)								
		*Serpina1*	(−0.93)												
														*Sting1*	(0.82)
														*Tf*	(0.80)
		*Tfrc*	(0.74)			*Tfrc*	(0.84)							*Tfrc*	(0.59)
		*Tnf*	(1.74)	*Tnf*	(0.76)			*Tnf*	(1.11)	*Tnf*	(1.45)				
**3 genes**		**8 genes**		**5 genes**		**6 genes**		**3 genes**		**5 genes**		**5 genes**		**10 genes**	

## Data Availability

The original contributions presented in this study are included in the article. Further inquiries can be directed to the corresponding author(s).

## Supplementary Materials

The following supporting information can be downloaded at: https://www.mdpi.com/article/10.3390/nano15171364/s1, Figure S1: Top 20 most frequently differentially expressed genes in mouse (A) and rat (B) experiments; Table S1: Number of significantly enriched pathways identified in lung transcriptomic analyses.
