# Supplementary material for: Comparison of Lung Inflammatory and Transcriptional Responses in Mice and Rats Following Pulmonary Exposure to a Fiber Paradigm-Compatible and Non-Compatible MWCNT
Source: Nanomaterials (Basel). 2025 Sep 4;15(17):1364. doi: 10.3390/nano15171364 (PMC12430210; doi:10.3390/nano15171364)
Supplement: Supplementary file 1 [file nanomaterials-15-01364-s001.zip › nanomaterials-3809623-supplementary.pdf]

Supplementary Materials

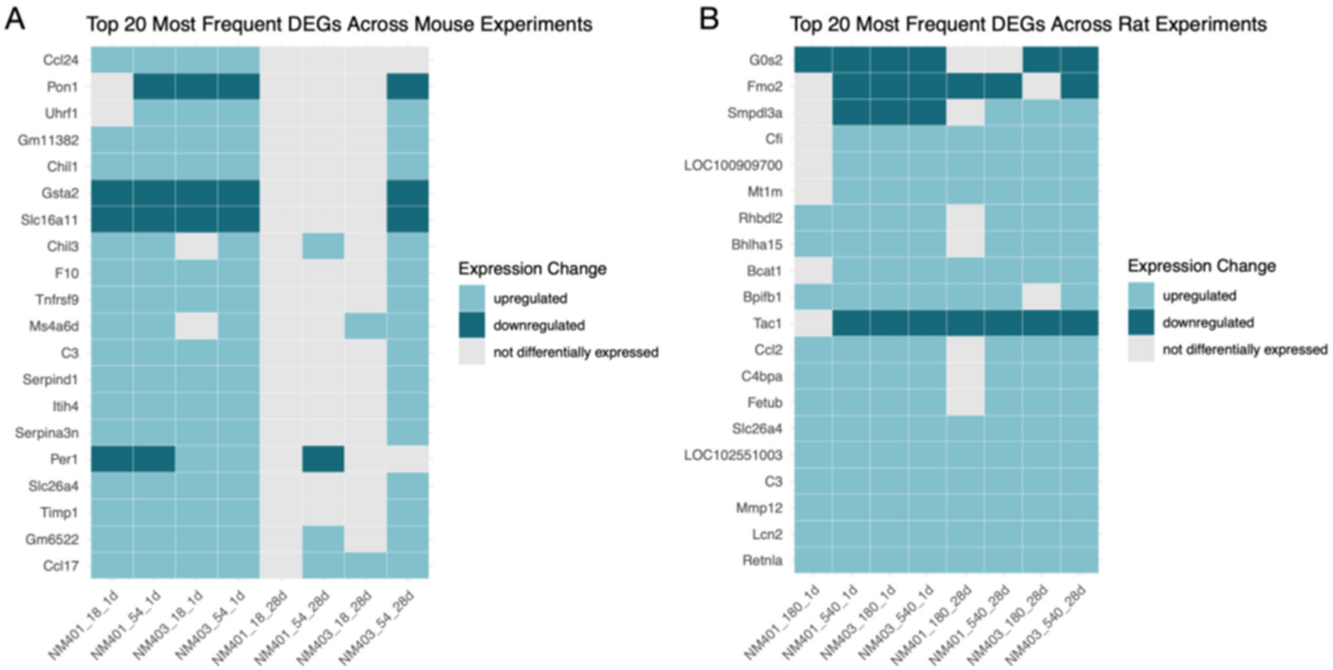

Figure S1. Top 20 most frequently differentially expressed genes in mouse (A) and rat (B) experiments. Colored tiles denote significant differential expression, with light blue associated with upregulation in the experimental condition while dark blue marks downregulation. Grey tiles indicate the gene was not significantly differentially expressed in the experimental condition.

Table S1. Number of significantly enriched pathways identified in lung transcriptomic analyses of mice and rats exposed to NM-401 and NM-403 at different doses and time points, along with totals and shared pathways across species.

**Experiment**	**significant pathways**
NM401_18_1d	2
NM401_54_1d	20
NM403_18_1d	11
NM403_54_1d	31
NM401_18_28d	0
NM401_54_28d	21
NM403_18_28d	9
NM403_54_28d	54
NM401_180_1d	7
NM401_540_1d	17
NM403_180_1d	11
NM403_540_1d	16
NM401_180_28d	3
NM401_540_28d	28
NM403_180_28d	17
NM403_540_28d	19
mouse total pathways at any time point or dose	78
rat total pathways at any time point or dose	56
shared between rat and mouse from all these	45
